# Supplementary figures and images for: Characterizing the Mechanism of Action of an Ancient Antimicrobial, Manuka Honey, against Pseudomonas aeruginosa Using Modern Transcriptomics
Source: mSystems. 2020 Jun 30;5(3):e00106-20. doi: 10.1128/mSystems.00106-20 (PMC7329319; doi:10.1128/mSystems.00106-20)

A

Color Key

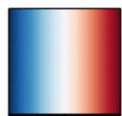

-2 2  
Log<sub>2</sub> FC

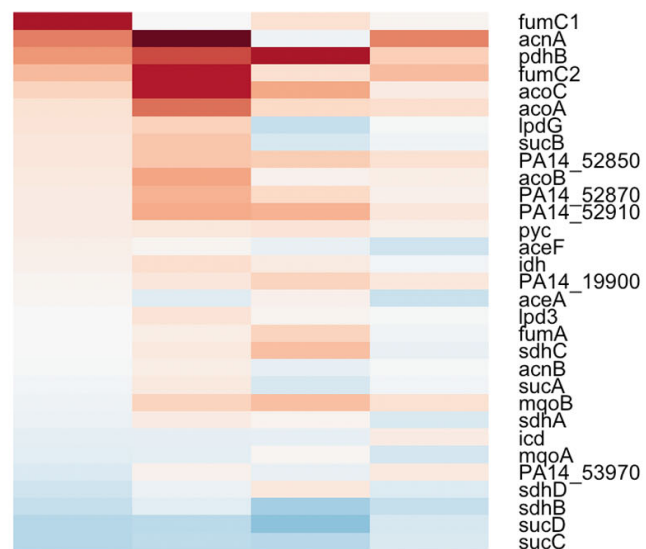

mh

ahmgo

mgo

ah

B

Color Key

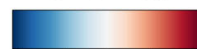

-4 0 2 4  
Log<sub>2</sub> FC

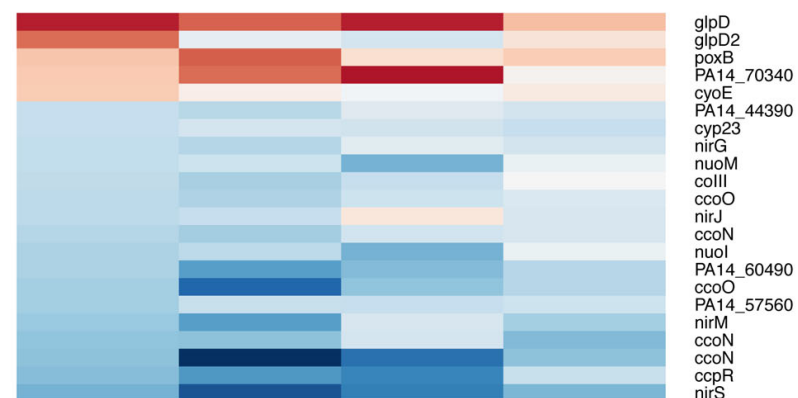

mh

ahmgo

mgo

ah

Supplement: FIG S2 [file mSystems.00106-20-sf002.pdf]

A

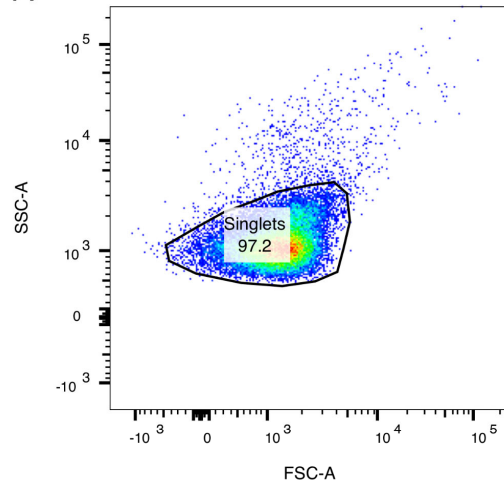

B

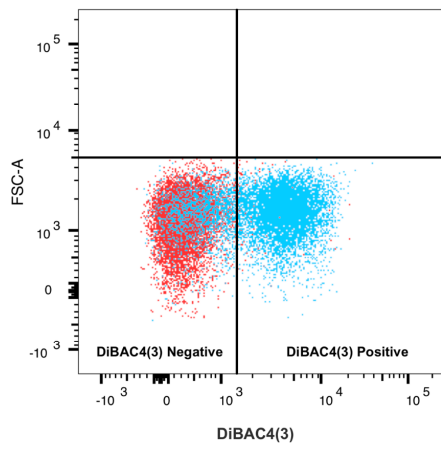

C

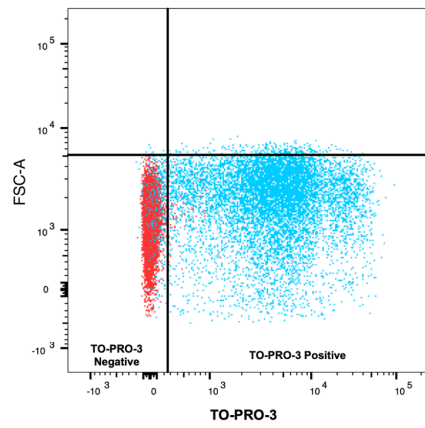

Supplement: FIG S3 [file mSystems.00106-20-sf003.pdf]
