# Supplementary material for: Characterizing the Mechanism of Action of an Ancient Antimicrobial, Manuka Honey, against Pseudomonas aeruginosa Using Modern Transcriptomics
Source: mSystems. 2020 Jun 30;5(3):e00106-20. doi: 10.1128/mSystems.00106-20 (PMC7329319; doi:10.1128/mSystems.00106-20)
Supplement: TABLE S1 [file mSystems.00106-20-st001.docx]

| **Strain** | **Relevant characteristics** | **Source or reference** |
| --- | --- | --- |
| *P. aeruginosa* |  |  |
| PAO1 | Wildtype | 1 |
| PA14 | Wildtype | 2 |
| PAO1Δ*recA* | *recA* deletion mutant of PAO1 | 3 |
| PAO1Δ*lys* | *lys* deletion mutant of PAO1 | 3 |
| PAO1Δ*prtN* | *prtN* deletion mutant of PAO1 | 3 |
| PAO1-LAC-EcPore | ‘hyperporinated’ PAO1, a chromosomally encoded modified *E. coli* FhuA siderophore uptake channel (EcPore) with an IPTG inducible promoter. | 4 |
| PAO1-LAC | Empty expression cassette | 4 |
| ΔPA14_03930 | PA14_03930::MAR2xT7 | 5 |
| ΔPA14_06510 | PA14_06510::MAR2xT7 | 5 |
| ΔPA14_18750 | PA14_18750::MAR2xT7 | 5 |
| ΔPA14_18760 | PA14_18760::MAR2xT7 | 5 |
| ΔPA14_18780 | PA14_18780::MAR2xT7 | 5 |
| ΔPA14_24940 | PA14_24940::MAR2xT7 | 5 |
| ΔPA14_32380 | PA14_32380::MAR2xT7 | 5 |
| ΔPA14_33520 | PA14_33520::MAR2xT7 | 5 |
| ΔPA14_33970 | PA14_33970::MAR2xT7 | 5 |
| ΔPA14_38040 | PA14_38040::MAR2xT7 | 5 |
| ΔPA14_38640 | PA14_38640::MAR2xT7 | 5 |
| ΔPA14_38660 | PA14_38660::MAR2xT7 | 5 |
| ΔPA14_51830 | PA14_51830::MAR2xT7 | 5 |
| ΔPA14_51430 | PA14_51430::MAR2xT7 | 5 |
| ΔPA14_51600 | PA14_51600::MAR2xT7 | 5 |
| ΔPA14_53290 | PA14_53290::MAR2xT7 | 5 |
| ΔPA14_58000 | PA14_58000::MAR2xT7 | 5 |
| ΔPA14_58030 | PA14_58030::MAR2xT7 | 5 |
| ΔPA14_58450 | PA14_58450::MAR2xT7 | 5 |
| ΔPA14_61020 | PA14_61020::MAR2xT7 | 5 |
| ΔPA14_62400 | PA14_62400::MAR2xT7 | 5 |
| ΔPA14_67500 | PA14_67500::MAR2xT7 | 5 |

References for Table S1

1. Jacobs MA, Alwood A, Thaipisuttikul I, Spencer D, Haugen E, Ernst S, Will O, Kaul R, Raymond C, Levy R, Chun-Rong L, Guenthner D, Bovee D, Olson MV, Manoil C. 2003. Comprehensive transposon mutant library of *Pseudomonas aeruginosa*. Proc Natl Acad Sci U S A 100:14339–14344. <https://doi.org/10.1073/pnas.2036282100>.
2. Rahme LG, Stevens EJ, Wolfort SF, Shao J, Tompkins RG, Ausubel FM.1995. Common virulence factors for bacterial pathogenicity in plants and animals. Science 268:1899 –1902. <https://doi.org/10.1126/science.7604262>.
3. Turnbull L, Toyofuku M, Hynen AL, Kurosawa M, Pessi G, Petty NK, Osvath SR, Carcamo-Oyarce G, Gloag ES, Shimoni R, Omasits U, Ito S, Yap X, Monahan LG, Cavaliere R, Ahrens CH, Charles IG, Nomura N, Eberl L, Whitchurch CB. 2016. Explosive cell lysis as a mechanism for the biogenesis of bacterial membrane vesicles and biofilms. Nat Commun 7:11220. <https://doi.org/10.1038/ncomms11220>.
4. Krishnamoorthy G, Leus IV, Weeks JW, Wolloscheck D, Rybenkov VV, Zgurskaya HI. 2017. Synergy between active efflux and outer membrane diffusion defines rules of antibiotic permeation into Gram negative bacteria. mBio 8:593. <https://doi.org/10.1128/mBio.01172-17>.
5. Liberati NT, Urbach JM, Miyata S, Lee DG, Drenkard E, Wu G, Villanueva J, Wei T, Ausubel FM. 2006. An ordered, nonredundant library of *Pseudomonas aeruginosa* strain PA14 transposon insertion mutants. Proc Natl Acad Sci U S A 103:2833–2838. <https://doi.org/10.1073/pnas.0511100103>.
