# Supplementary material for: Characterizing the Mechanism of Action of an Ancient Antimicrobial, Manuka Honey, against Pseudomonas aeruginosa Using Modern Transcriptomics
Source: mSystems. 2020 Jun 30;5(3):e00106-20. doi: 10.1128/mSystems.00106-20 (PMC7329319; doi:10.1128/mSystems.00106-20)
Supplement: TABLE S2 [file mSystems.00106-20-st002.docx]

| **Treatment** | **0.5 × MIC (% w/v)** | **MGO content (ppm)** |
| --- | --- | --- |
| Manuka honey | 5 | 45 |
| MGO | 27.5 | 247.5 |
| AHMGO | 10.75 | 96.75 |
| AH | 12.5 | 0 |
