# Supplementary material for: Characterizing the Mechanism of Action of an Ancient Antimicrobial, Manuka Honey, against Pseudomonas aeruginosa Using Modern Transcriptomics
Source: mSystems. 2020 Jun 30;5(3):e00106-20. doi: 10.1128/mSystems.00106-20 (PMC7329319; doi:10.1128/mSystems.00106-20)
Supplement: TABLE S3 [file mSystems.00106-20-st003.docx]

| Strain | Gene Name | MIC (% (w/v)) |
| --- | --- | --- |
| Wild-type UCBPP-PA14 |  | 10 |
| ΔPA14_03930 * | ΔspuE | 9 |
| ΔPA14_06510 * | ΔbioF | 9 |
| ΔPA14_18750 | ΔgloA1 | 9 |
| ΔPA14_18760 | ΔmexE | 8 |
| ΔPA14_18780 | ΔmexF | 8 |
| ΔPA14_24940 * |  | 9 |
| ΔPA14_32380 | ΔoprN | 8 |
| ΔPA14_33520 * |  | 8 |
| ΔPA14_33970 * |  | 9 |
| ΔPA14_38040 | ΔcmrA | 10 |
| ΔPA14_38640 | ΔscoB | 8 |
| ΔPA14_38660 | ΔscoA | 9 |
| ΔPA14_51830 | ΔpsqE | 8 |
| ΔPA14_51430 | ΔpqsA | 8 |
| ΔPA14_51600 * |  | 9 |
| ΔPA14_53290 | ΔtrxB2 | 8 |
| ΔPA14_58000 * | ΔsodM | 9 |
| ΔPA14_58030 * | ΔfumC | 8 |
| ΔPA14_58450 | ΔdppC | 9 |
| ΔPA14_61020 * |  | 8 |
| ΔPA14_62400 * |  | 9 |
| ΔPA14_67500 | ΔgloA3 | 5 |
| Wild-type PAO1 |  | 8.5 |
| PAO1Δlys * | Δlys | 8.5 |
| PAO1ΔrecA | ΔrecA | 7 |
| PAO1ΔprtN * | ΔprtN | 8.5 |

* indicates genes which were only differentially expressed (log_2_FC ≥ ±2, p.adj ≤ 0.05) in cells treated with manuka honey
