# Supplementary material for: Characterizing the Mechanism of Action of an Ancient Antimicrobial, Manuka Honey, against Pseudomonas aeruginosa Using Modern Transcriptomics
Source: mSystems. 2020 Jun 30;5(3):e00106-20. doi: 10.1128/mSystems.00106-20 (PMC7329319; doi:10.1128/mSystems.00106-20)
Supplement: TABLE S4 [file mSystems.00106-20-st004.docx]

| **Primer** | **Description** | **Sequence (5’ to 3’)** |
| --- | --- | --- |
| PA14_00710_F | *osmC* | GGCTTCAATACCCGCTTC |
| PA14_00710_R | *osmC* | GGCAGTGATGGCGAAAC |
| PA14_01710_F | *ahpC* | GGCAAGTTCATCGAGGTG |
| PA14_01710_R | *ahpC* | GTTTCGTGCCAGACCTTG |
| PA14_17060_F | *rpsB* | GACCTGGAAACCCAGTCC |
| PA14_17060_R | *rpsB* | CCCAGCTTGTTGGCTTC |
| PA14_17530_F | *recA* | CAGATCGAACGCCAATTC |
| PA14_17530_R | *recA* | ATCACCGAGAGGGTCAGG |
| PA14_25160_F | *lexA* | GCCGAACAGAACATCGAG |
| PA14_25160_R | *lexA* | TTTCACCGTGACCTCCTC |
| PA14_27220_F | *ohr* | TACTCGGCCTGCTTCATC |
| PA14_27220_R | *ohr* | TAGGGGCAGACCTGGTG |
| PA14_32390_F | *mexF* | AACCAGGGCTACGAGGAG |
| PA14_32390_R | *mexF* | TAGACCTGCAGGGTGTCG |
| PA14_37710_F | *fusA2* | CACCGCTATCGCTTCAAC |
| PA14_37710_R | *fusA2* | CGGTCCATCTTGTTCACG |
| PA14_48700_F | *kefB* | TGACTGTGCTGCTGATCG |
| PA14_48700_R | *kefB* | GGTGATGGAAAGCACCAG |
| PA14_49710_F | *yedU* | ACGAGATCTGCGTGTTCC |
| PA14_49710_R | *yedU* | TTGCCAAGGTTGTTGGAG |
| PA14_51430_F | *pqsA* | GCGGTTCTGGTTCCTACC |
| PA14_51430_R | *pqsA* | AACTTGCCGTTGTCGTTG |
| PA14_53290_F | *trxB2* | CCAGTTGACCACCACCAC |
| PA14_53290_QR | *trxB2* | AGTGCGTCGCAGGTGTAG |
| PA14_55130_QF | *gloA2* | TGGAGTTGACCCACAACTG |
| PA14_55130_QR | *gloA2* | TCTGGATCAGTTCCACCTTG |
| PA14_55610_QF | *dnaE2* | TGAGCGATTACGCTCTCC |
| PA14_55610_QR | *dnaE2* | TCCAGGGTGATGAAGGTG |
| PA14_56780_QF | *sodB* | GGGTACCGAGTTCGAAGG |
| PA14_56780_QR | *sodB* | TGTCGAAGGAGCCGAAG |
| PA14_60830_QF | *mexD* | CATCGGCTACGAGTGGAC |
| PA14_60830_QR | *mexD* | ATACCCGCGACCATTACC |
| PA14_61040_QF | *katB* | CAACCTCGACGACGACTC |
| PA14_61040_QR | *katB* | CTGCAGGCTCTTCCAGTG |
